# On the Metal Cofactor in the Tyrosinase Family

**DOI:** 10.3390/ijms19020633

**Published:** 2018-02-23

**Authors:** Francisco Solano

**Affiliations:** Department Biochemistry and Molecular Biology B and Immunology, School of Medicine and LAIB-IMIB, University of Murcia, 30100 Murcia, Spain; psolano@um.es; Tel.: +34-868-88-7194

**Keywords:** tyrosinase-related proteins, tyrosinase, melanin biosynthesis, copper, zinc, metal enzymes, acquisition protein structure

## Abstract

The production of pigment in mammalian melanocytes requires the contribution of at least three melanogenic enzymes, tyrosinase and two other accessory enzymes called the tyrosinase-related proteins (Trp1 and Trp2), which regulate the type and amount of melanin. The last two proteins are paralogues to tyrosinase, and they appeared late in evolution by triplication of the tyrosinase gene. Tyrosinase is a copper-enzyme, and Trp2 is a zinc-enzyme. Trp1 has been more elusive, and the direct identification of its metal cofactor has never been achieved. However, due to its enzymatic activity and similarities with tyrosinase, it has been assumed as a copper-enzyme. Recently, recombinant human tyrosinase and Trp1 have been expressed in enough amounts to achieve for the first time their crystallization. Unexpectedly, it has been found that Trp1 contains a couple of Zn(II) at the active site. This review discusses data about the metal cofactor of tyrosinase and Trps. It points out differences in the studied models, and it proposes some possible points accounting for the apparent discrepancies currently appearing. Moreover, some proposals about the possible flexibility of the tyrosinase family to uptake copper or zinc are discussed.

## 1. Introduction

**Figure 1:**
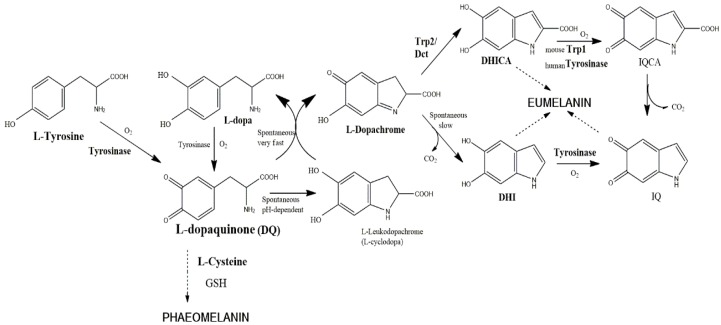
The currently assumed Raper–Mason pathway for eumelanin formation in mice. The precursor, l-tyrosine, is oxidized by molecular oxygen to form l-dopaquinone. The reaction is catalyzed by tyrosinase and is comprised of two stages, hydroxylation of monophenol (tyrosine) to *o*-diphenol (DOPA) and oxidation of this intermediate to *o*-l-dopaquinone. In the presence of thiol-free compounds (e.g., l-Cys, glutathione), sulfur-containing pheomelanin is formed. In the absence of such compounds, l-dopaquinone undergoes an internal cyclization to l-leucodopachrome (l-cyclo-DOPA). The coexistence of the last two derivatives is unstable, as they react in a very fast redox disproportion to regenerate l-DOPA and l-dopachrome. l-DOPA is oxidized by tyrosinase, and l-dopachrome evolves spontaneously to 5,6-dihydryindole (DHI) by a decarboxylative rearrangement, unless the presence of the enzyme Trp2 (dopachrome tautomerase) catalyzes the non-decarboxylative rearrangement to 5,6-dihydryindole-2-carboxylic acid (DHICA). DHI and DHICA are *o*-diphenols susceptible to new oxidations to the corresponding indolequinones by specific action of tyrosinase and Trp1, respectively. Human tyrosinase seems to be able to oxidize both DHI and DHICA, so that human Trp1 does not have a well-demonstrated role (see the text). Dihydroxyindoles and indolequinones spontaneously cross-link to form oligomers and eumelanin polymer.

Until around 1980, it was believed that tyrosinase was the unique enzyme required for melanogenesis in all types of cells [1,2]. Melanin pigmentation is a widely-occurring protective process in all types of cells against radiations (UV light and others), oxidative stress and environmental harmful conditions [3]. Once tyrosinase catalyzes the formation of an *o*-quinone from an *o*-diphenol, the subsequent reactions until melanin can occur spontaneously. Actually, bacteria, fungi and plants contain a unique enzyme to trigger the melanin biosynthetic pathway. This enzyme is usually called tyrosinase in microorganisms and catechol oxidase in plants. Tyrosinase enzyme (EC 1.14.18.1) catalyzes the first two steps of the Raper–Mason pathway, the hydroxylation of l-tyrosine to l-DOPA and the subsequent oxidation of l-DOPA to l-dopaquinone (Figure 1). The first activity is called tyrosine hydroxylase, and the second one *o*-diphenol oxidase, catechol oxidase or DOPA oxidase. Both activities cannot be separated. However, in plants, the enzyme cannot catalyze the hydroxylation, and it only shows the second activity. In addition, it also shows low specificity for the *o*-diphenolic substrate, so that plant tyrosinases are able to oxidize a number of catechol or polyphenols occurring in the vegetal tissues. In that way, the names catechol oxidase or polyphenol oxidase have been preferred to tyrosinase [4]. Nevertheless, recently, some plant catechol oxidases have been described showing tyrosine hydroxylase activity [5,6], confirming that tyrosinases and catechol oxidases are very similar enzymes with subtle differences at the active site. They belong to the type 3 copper oxidases, as hemocyanins and even laccases [7,8,9,10]. In any case, after dopaquinone or any other *o*-quinone is formed by the oxidative action of these enzymes, the pathway progresses by a series of spontaneous reactions to lead to the final melanin, a structurally-ill-defined polymer [3].

In animal melanocytes, all steps after l-dopaquinone formation were also thought to proceed spontaneously, as “in vitro” l-dopaquinone solutions are unstable, and this *o*-quinone evolves to melanin at room temperature and neutral pH in a few hours. However, around 1980, a number of growing pieces of evidence indicated a lack of correlation among tyrosinase activity, melanin formation in animal skin and hair and blood levels of melanocortin, the animal hormone to control the melanogenic capacity of melanocytes [11,12]. The accumulation of such evidence launched the possibility that the hormonal control of melanogenesis acted on other proteins involved in mammalian melanogenesis. Soon, data strongly suggested the existence of a post-tyrosinase regulation in this process beyond l-dopaquinone or l-dopachrome formation [1,2,13,14,15]. 

Thus, in animals, the tyrosinase family of proteins is composed of three members, the authentic tyrosinase and two other related proteins, named Trp1 and Trp2. Studies on a rare ascidian organism, sea pineapple, indicate that triplication of the tyrosinase family occurred during the early adaptive radiation of chordates. Initially, duplication of an ancestral tyrosinase gene produced a single *TRP* gene before the cephalochordate-vertebrate divergence, and a new subsequent duplication of the ancestral *TRP* gene gave rise to *TRP1* and *TRP2* genes before the emergence of teleost fishes [16]. Hence, these three genes are found in all animals, and they play a crucial role in determining melanin production and coloration of animal pigment cells.

The three members of the family show a common architecture of the tridimensional structure, and the three proteins are anchored to the melanosomal membrane through a C-terminal fragment. They show approximately 40% amino acid identity and the same structural features, including a signal peptide, a Cys-rich domain, two metal binding sites and a transmembrane domain. The Cys-rich domain is composed of an “EGF-like” region and another Cys-rich fragment at the central part of the sequence, between the two metal binding sites. The bulk of the protein is a globular intramelanosomal domain followed by a single transmembrane fragment and a small C-terminal tail oriented to the cytosol of melanocytes [15,17].

The intramelanosomal domains of the three proteins are similar in length, and they contain a binuclear metal ion-binding motif with six conserved His [18]. However, the C-terminal tails show very low homology, and they were used to generate specific antibodies, PEP1, PEP7 and PEP8 for Trp1, tyrosinase and Trp2, respectively [15,19]. The specific reactivity of those antibodies against each C-tail allowed the differentiation, characterization and quantitation of the three proteins.

### 1.1. Cloning and Characterization of the Animal Tyrosinase Gene Family

In the past century, around the second half of the 1980s, the preparation of cDNA and the cloning techniques became easily available. These techniques were applied to explore the melanogenic system in mouse and human. Trp1 was the first melanogenic enzyme whose gene was cloned from a mouse gene library [20], and in principle, it was believed to be the cloning of the tyrosinase gene.

However, the authentic mouse tyrosinase was cloned soon after [21,22], as well as human tyrosinase [23]. According to that, it was proposed that two tyrosinases from two different loci were expressed by normal and by transformed melanocytes [19], mapped to the albino (c) and brown (b) loci, and both seemed to have the catalytic functions ascribed to tyrosinase, i.e., hydroxylation of l-tyrosine to l-DOPA and the oxidation of l-DOPA to l-dopaquinone. This confirmed that regulation of melanogenesis in mammals was more complex than in lower organisms, and it was controlled by more than one enzyme. It was determined that the specific activity of the protein encoded by the *albino locus* gene was considerably higher than that of the protein encoded by the *brown locus*, but the brown protein was present in melanocytes in a higher amount than is the protein encoded by the *albino locus*. The alternative names protein b and gp75 (glycoprotein of a 75-Kd molecular mass) were also used for Trp1 protein. The sequence of the human *TRP1* gene was also published [24], and it was found to be regulated by a promoter sequence very different from the tyrosinase promoter [25] accounting for the different expression of both proteins in melanocytes.

A third member of the tyrosinase family was soon cloned, in mouse [26] and human [27]. This member also showed around 40% homology with the other two proteins of the family, and it was called Trp2. The murine enzyme mapped at the *slaty locus.* This was the last member of the family, as another possible fourth candidate, the product of the *silver locus* (Pmel17), was tentatively assigned to the tyrosinase family [28], but soon excluded as Pmel17 is a melanosomal matrix structural protein devoid of enzymatic activity and without homology to the tyrosinase family.

### 1.2. On the Function of Trp2 and Trp1

The *TRP2* gene product was soon assigned to the enzyme dopachrome tautomerase, which catalyzes the conversion of dopachrome to 5,6-dihydroxyindole-2-carboxylic acid (DHICA) [14,17,29]. Tautomerization is a type of isomerization rearranging dopachrome to DHICA rather than to DHI (5,6-dihydroxyindole), which is generated spontaneously by decarboxylation (Figure 1).

That branch point in the eumelanin biosynthetic pathway introduced important implications as regulatory control. The two dihydroxyindoles show different stability in the culture medium, DHI being less stable and more toxic than DHICA [30]. The cytotoxicity of DHI has been exploited as a targeting concept in experimental melanoma therapy. The presence of Trp2 leads to an increase in the ratio of DHICA/DHI that would prevent that cytotoxicity and premature cell death [31]. Thus, Trp2 is a protective enzyme for melanocytes to minimize the cytotoxic effects of DHI, rather than an enzyme to enhance the amount of pigment formed.

The role of Trp1 in melanogenesis has remained elusive from its discovery, with controversial results described by different groups. Firstly, as this protein is usually more abundant than authentic tyrosinase in mammalian melanocytes, it was considered as a second tyrosinase with low specific activity [19] for tyrosine hydroxylation and DOPA oxidation. However, the transient expression of its cDNA in non-melanocytic cells and assays on the purified human protein [32] failed to substantiate this enzymatic activities. Concomitant with these investigations, Trp1 was reported as a catalase to enhance the formation of eumelanin by hydrogen peroxide decomposition [33]. Due to its interaction with tyrosinase, it was also proposed that Trp1 was rather a stabilizing protein for the authentic tyrosinase with doubtful enzymatic activity [15,34]. However, the most accepted function attributed to Trp1 was the DHICA oxidase activity needed after Trp2 action in the distal phase of melanogenesis [35,36].

On the other hand, Winder et al. [37] analyzed the activities of tyrosinase and Trp1 by expression of these proteins in fibroblasts. They detected some DOPA oxidase activity in tyrosinase-expressing cells, but they did not confirm catalase, DOPA oxidase or DHICA oxidase activity in extracts of fibroblasts expressing Trp1. Unexpectedly, they detected dopachrome tautomerase activity. Moreover, culture media from the cell line expressing both proteins, tyrosinase and Trp1, contained significant amounts of 6-hydroxy-5-methoxyindole-2-carboxylic acid, which would be expected in cells with active dopachrome tautomerase [38]. Hence, they concluded that Trp1 could act as a dopachrome tautomerase in vivo, as well as in vitro.

As this activity, was clearly assigned to Trp2 and the reasons why melanocytes would contain two distinct enzymes with dopachrome tautomerase enzymes were unclear, the findings of Winder et al. [37,38] have rarely been considered. On the other hand, the DHICA oxidase activity of Trp1 is not totally clear and depends on the species. It has been reported that the human enzyme does not show DHICA oxidase activity in contrast to murine Trp1 [39]. The DHICA oxidase activity in human melanocytes was later attributed to tyrosinase [40]. Some recent reports about crystallized human Trp1 (see below) suggest that the data described by Winder et al. [37,38] more than 20 years ago should be reconsidered.

### 1.3. Metal Proteins

Around 30% of all folded proteins coordinate a metal ion to get their physiological function [41]. The binding of the metal normally affects the stability and physical properties of the protein. Understanding how metals are utilized by proteins in cells on a molecular level frequently requires accurate descriptions of the thermodynamic and kinetic parameters involved in protein-metal complexes. There are examples of metal binding before, during or after folding of the polypeptide [42]. The involvement of the metal ions in the catalytic action of metal enzymes is also usual, and they occupy essential positions at the active site. This implies that the metal ions should be coordinated to the active site during protein maturation before reaching the final destination.

There is no doubt that tyrosinase contains copper ions at the catalytic center. The first clear evidence of the copper-enzyme nature of mammalian tyrosinase was reported as earlier as 1950 [43]. All tyrosinases, including bacterial tyrosinases and plant polyphenol oxidases, contain a pair of copper ions at the flexible active site [44]. These enzymes belong to the type-3 copper-protein class, and they are currently being studied intensively [5,6,9,45,46]. These sites comprise six histidine residues that coordinate the two copper ions CuA and CuB. They are conserved among tyrosinases, catechol oxidases and hemocyanins. These copper ions bind an oxygen molecule and they show redox properties during the catalytic cycle involving cupric and cuprous forms, although other copper states are possible during enzyme inactivation [47].

Tyrosinase and Trps share many common structural features, including the two metal binding sites essential to their catalytic functions. Concerning the nature of the metal cofactor in Trps, the presence of Zn(II) ions at Trp2 seems to be clearly demonstrated. This was the first exception of a protein showing features of the type-3 copper proteins, but loading an alternative ion. In fact, Trp2 was first described as an iron-binding enzyme [48], but reconstitution of dopachrome tautomerase activity after incubation of different cations with the apo-protein indicated that Trp2 was a Zn(II) enzyme [49]. The involvement of Zn(II) instead of Fe(II) is also in agreement with the non-redox, but tautomerase activity of this enzyme [50].

The situation about the metal cofactor of Trp1 is not so clear. The first data for gp75, the older name of Trp1 prior to gene cloning, suggested that the metal might be iron due to the catalase activity attributed to that protein [33]. A direct study using radiolabeled metal ligand binding for reconstitution followed by autoradiography demonstrated that tyrosinase binds copper, Trp2 binds zinc, but Trp1 does not bind copper, zinc or iron under the experimental conditions used [51]. Thus, the presence of Cu(II) in Trp1 was not definitely proven. According to its DHICA oxidase activity and similarities to tyrosinase, it has been accepted that copper would be the most likely metal cofactor. Tyrp1 would be a second poor tyrosinase with residual DOPA oxidase activity and, at least in the case of the mouse enzyme, a DHICA oxidase [35,36,40]. However, experiments using chimeric constructs of tyrosinase and Tyrp1 composed of a first part with the CuA binding site of tyrosinase and a second part with the likely CuB binding site of Trp1 did not show any enzymatic activity [52].

### 1.4. Intracellular Availability of Copper and Zinc

Maintenance of metal homeostasis, mainly iron, copper and zinc, is crucial for many different enzymatic activities and in turn for cell function and survival. Copper is one of the transition metals frequently found at the active sites of many enzymes mostly involved in enzymatic redox catalysis. Copper can act as both an antioxidant and a pro-oxidant, although pro-oxidant actions are much more prevalent. As an antioxidant, Cu scavenges or neutralizes free radicals and may reduce cell damage. When copper acts as a pro-oxidant, it promotes free radical damage [53]. The amount of intracellular free copper must be strictly limited to very low levels because of its toxic effects [54]. Thus, all cells have several systems for copper trafficking and copper storage in cellular compartments [55]. Although these systems are not totally understood, there are some data available on the structure and mechanism of copper transference [56]. Thermodynamic data suggest that copper is drawn to the target proteins that require it with the involvement of chaperones containing copper binding sites. This implies protein-protein specific recognitions exploiting gradients of increasing copper-binding affinity. Metallothioneins have the highest affinity for copper ion and may play special roles in the regulation of cellular copper distribution. Other proteins, such as the Menkes protein, are required for Cu(II) transfer to human tyrosinase [57].

Zn biochemistry gained importance after the discovery of zinc-finger proteins. The human genome encodes around 3000 zinc proteins. Zinc is the second most abundant transition metal in organisms after iron, and it is the only metal that appears in all enzyme classes as a catalytic or structural cofactor, so that the biological impact of zinc is at least as important as that of copper and iron [58]. There are some proteins controlling the homeostasis, vesicular storage, concentration and subcellular distribution of Zn(II), but in lower amounts than copper, as zinc does not show oxidant properties and is far less toxic. Thus, the intracellular availability of Zn(II) ions for unspecific incorporation with proteins is higher than that of Cu(II) in the cytosol, as well as cellular compartments.

It seems that Zn and Cu do not share mechanisms for trafficking and incorporation with the proteins. Even in the case of copper-zinc superoxide dismutase, two transfer pathways have been identified for copper incorporation involving a copper chaperone and glutathione [59], but both mechanisms are specific for copper and are not shared for Zn incorporation.

It is clear that Cu(II) promotes pigmentation in the skin, hair and eyes of animals, as it is needed for the tyrosinase activity. In addition to that, Cu(II) ions accelerate the conversion of dopachrome to DHICA and DHI and its subsequent oxidation to the melanin polymer [60]. Zn(II) also modulates melanogenesis, but the net effect is unclear. Zn(II) inhibits tyrosinase in vitro, but also enhances dopachrome tautomerase activity (Trp2). It is well documented that oral ingestion of Zn(II) affects the degree of pigmentation and the melanosome structure. High doses of zinc sulfate inhibit eumelanogenesis and cause severe murine hair hypopigmentation [61]. On the other hand, low zinc diets produce abnormal large melanosomes in choroidal melanocytes of adult pigs resulting in unusual and aberrant melanin distribution [62]. The balance of copper to zinc is perhaps more important than the concentration of either of these ions. Some fast copper/zinc exchange reactions have been described in disordered proteins with important roles in neurodegenerative diseases [63], but these exchanges are unlikely in enzymes with a defined native structure. The first ion that occupies the enzyme-binding site remains on it unless it is removed by incubation with very high-affinity chelators to prepare apoenzyme.

### 1.5. Tyrosinase Models for Trps: Consequences of the Metal Cofactor

In the last decade, some structural models of crystallized tyrosinases and catechol oxidases from bacteria, fungi and plants have been reported [8,44,64,65]. However, the difficulty of isolating pure and homogeneous tyrosinase, Tyrp1 and Tyrp2 from mammalian melanocytes have hampered the crystallization of these enzymes. However, recently, powerful techniques for the heterologous expression in insect cells of the bulky part of the proteins have allowed the availability of human tyrosinase [66] and Trp1 [67], and the tridimensional data on these two proteins have become recently available for the first time. This should be a huge advance to understand the features of the mammalian tyrosinase family and its respective role in the mammalian melanosome. It can also improve some remaining unconcluded questions in the mechanisms of action of the tyrosinase family, such as the differences among monophenol hydroxylase and o-diphenol oxidase activities or between tyrosinases and catechol oxidases looking for key residues determining the different affinities of both groups [5,6,8,9,45]. However, one of the most striking points from the structural point of view is the achievement of crystallized species of mammalian Trp1 containing Zn(II) instead of the expected Cu(II) [67,68]. That would make it unlikely that human Tyrp1 acts as a redox enzyme showing DHICA oxidase activity.

**Figure 2:**
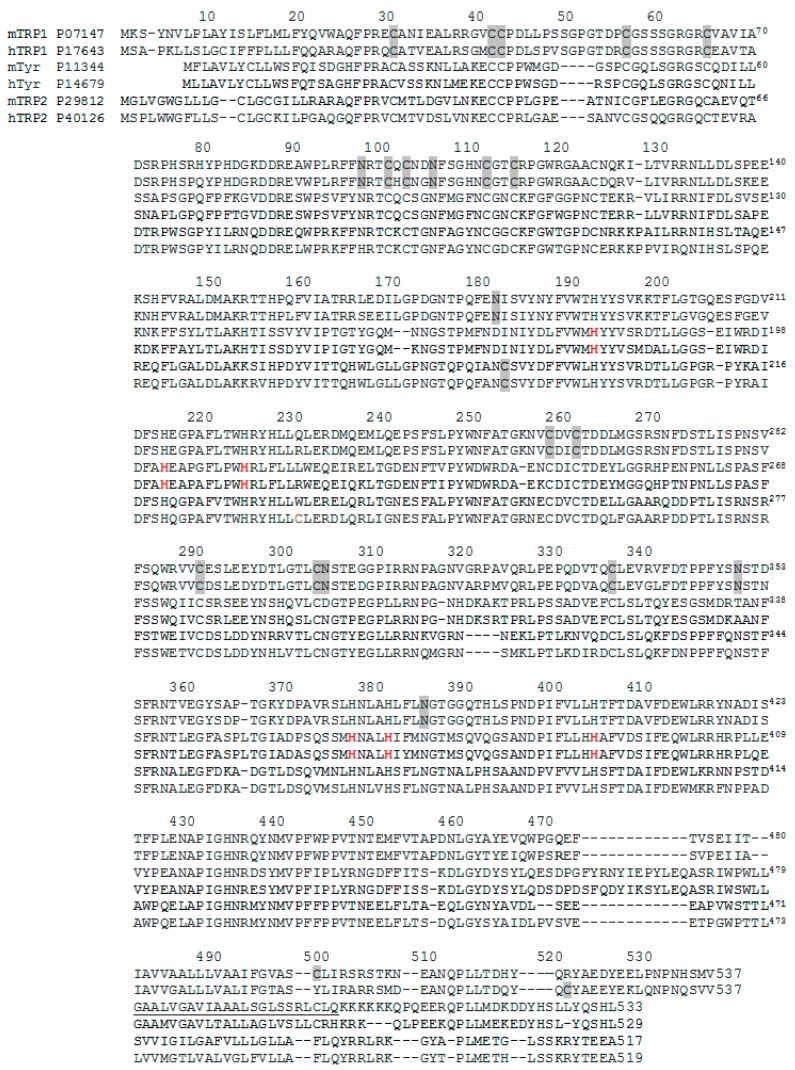
Sequence alignment of mouse (m) and human (h) Trp1 (first two lines), tyrosinase (third and fourth) and Trp2 (fifth and sixth). Alignment was performed with Expasy facilities. References of the six proteins are indicated in the initial line for each one taken from the UniProtKB database (Available online: www.expasy.org, [69]). Important Cys and Asn residues are indicated on Trp1 sequences (grey residues). Conserved His at the MeA and MeB binding sites are marked in bold on the tyrosinase sequence (central lines). The transmembrane fragment is underlined in mouse tyrosinase. For other details and relevant residues, see the text.

The Zn-enzyme nature would be in agreement with the reported inability of human Trp1 to show DHICA oxidase activity [39], but the mouse enzyme does show that activity [35,36]. This situation would lead to the proposal that human Trp1 binds Zn(II), but mouse Trp1 binds Cu(II) at the active site. Amino acid variations between human and mouse Trp1 do not justify this important functional difference (Figure 2), although some amino acids are still marked as “sequence conflict” in protein databases [69]. A very recent smart paper by Decker et al. [70] states that the important findings of Lai et al. [67,68] on human Trp1 solve a problem, but it creates some new ones concerning enzymatic specificities of the copper-3 enzymes to other metal ions. Trp2 has the motif of the copper-3 enzymes, but it binds zinc instead of copper. 

The binding of Zn(II) ions to the active site of the tyrosinase family is not surprising. In addition to Trp2 [49,50], there are a number of papers reporting that tyrosinase and Trp1 can bind Cu(II), and also Zn(II) [8,71,72] under appropriate conditions, mostly the presence of Zn(II) and low availability of Cu(II). Replacement of Cu(II) by Zn(II) induces a conformational change in the protein [71]. In crystallized tyrosinase from *Bacillus megaterium*, both monophenol hydroxylase and o-diphenol oxidase activities are lost, suggesting that Zn(II) binding likely competes with Cu(II) at the His-active site.

At this stage, the data about the dopachrome tautomerase activity of Trp1 when this gene was transfected in fibroblasts should be recapitulated [37,38]. Unfortunately, the metal bound to that Trp1 was not determined, but the most plausible assumption is that the metal might is Zn(II). This would mean that human and also mouse Trp1 are able to bind Zn(II) and behave as a second dopachrome tautomerase under conditions favoring Zn(II) incorporation. In addition, Cu-Trp1 showing a certain DHICA oxidase activity can also be obtained [68], according to the reported function for this enzyme [35,36]. All together, these results suggest an attractive hypothesis: Trp1 could bind Cu(II) or Zn(II), depending on the conditions, mimicking a second tyrosinase or a second dopachrome tautomerase, respectively. Metal-proteins usually show a certain specificity for the appropriate metal cofactor, but one metal ion may be replaced by another similar one. In vitro incubations of apo-tyrosinase with other metal ions differing Cu(II) result in metal-proteins with very low or null enzymatic activity [72,73]. Trp1 could also undergo this ion exchange, but keeping enzymatic activities and cellular significance. Exchanging Zn(II) by Cu(II) conferred some DHICA oxidase activity to Trp1 [68]. Moreover, the triple change Y362F, R374S T391V in Trp1-3M, mimicking three positions of tyrosinase, has no effect on its enzymatic properties. This agrees with negative results about the recovery of tyrosinase activity using constructions of chimeric species with half of mouse tyrosinase and half of mouse Trp1 [52].

Another important aspect about the likely dopachrome tautomerase activity of Zn-Trp1 would be the specificity for the substrate. Purified Trp2 shows stereospecifically on l-dopachrome, and the reaction is non-decarboxylative, leading to DHICA as the unique product [29]. This enzyme has each Zn(II) bound to the protein moiety of the enzyme through three His residues conserved in the metal sites MeA and MeB, probably forming a distorted tetrahedron, the usual geometry of this ion in Zn-proteins. The fourth position would be occupied by an exogenous ligand, probably water [50]. Some other residue(s) should interact with the carboxyl group of dopachrome to account for the specificity for l-dopachrome, but the identity of these residues is unknown.

On the other hand, other non-specific reactions on dopachrome-like quinones have been reported. For instance, Trp2 expressed in HEK cells significantly reduced the sensitivity of these cells to dopamine and hydroquinone [31], suggesting that when the enzyme is expressed in other non-melanocytic cells, it is able to act on quinone metabolites other than dopachrome, as dopaminechrome forming from dopamine. There is also a number of interesting reports about Trp2-related enzymes in lower organisms, such as cuttlefish [74], which are able to act on l- or d-dopachrome and also on the decarboxylated dopaminechrome to give DHI. Interestingly, the homologue enzyme to mammalian Trp2 in insects has been widely studied. That enzyme catalyzes the decarboxylation of dopachrome, and it is also able to act on dopaminechrome [75,76]. Related to that, human Zn-Trp1 species expressed in insect cells could act similarly to native Trp2 enzyme in those organisms, as it has been reported that it does not show any interaction with the carboxyl group of l-dopachrome [67,68].

### 1.6. Some Other Considerations about Factors Affecting the Structure of the Tyrosinase Family

Undoubtedly, the results reported by Lai et al. [67] about Zn(II) binding to human Trp1 in the insect system used for heterologous expression show reliability. They produced tyrosinase and Trp1 under identical cell culture conditions, and the two proteins consistently contain different ions, Cu(II) in tyrosinase and Zn(II) in Trp1. However, the finding of Zn-Trp1 species raises some uncertain factors about the real existence of such species in melanocytes and the actual role of the entire protein in melanogenesis. It should be taken into account that the Zn(II)-Trp1 species studied do not comprise the entire protein, but the intramelanosomal portion comprising positions 25–471. That protein comprises the native sequence of human Trp1 up to position 471, followed by a tobacco etch virus (TEV) cleavage site and a hexa-His tag [67]. Thus, the transmembrane fragment and the important C-terminal tail of the entire melanosomal protein are missing in the insect cells.

**Figure 3:**
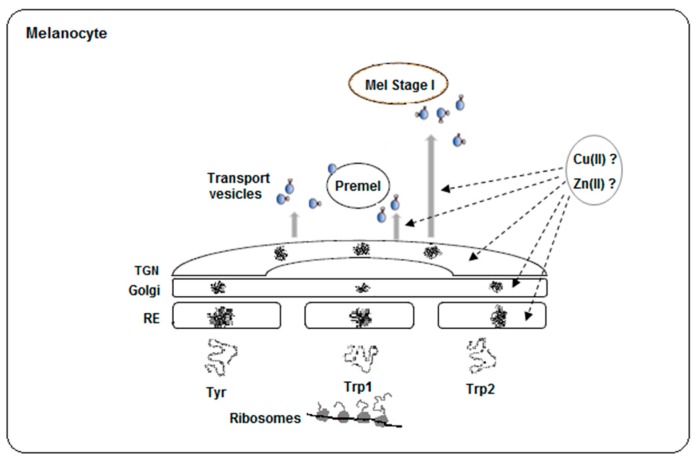
Scheme of the traffic and maturation of the tyrosinase family. Nascent polypeptides coming from polyribosomes pass through endoplasmic reticulum (ER), Golgi and TGN (trans-Golgi network) for proper folding. Then, *N*-glycosylated proteins are transported to premelanosomes or melanosomes in vesicles by recognition of specific adaptor proteins to motifs at the respective C-carboxy tails. Truncation of the C-terminal may alter the pathway of the proteins. On the other hand, the location for Zn(II) or Cu(II) acquisition by apo-proteins is unknown, but different traffic might imply different metal incorporation. Experimental data indicate that the metal binding site in the tyrosinase family seems to accommodate both ions.

As stated above, tyrosinase is the member of the family most widely studied concerning all features, including processing, maturation and metal incorporation (Figure 3). The mechanisms for copper incorporation to tyrosinase are variable throughout nature. They involve that of a caddie protein in some bacteria to complex specific chaperones in animal melanocytes. Eukaryotic cells transfer different metal ions to proteins in different compartments [42,54]. In melanocytes, copper incorporation in tyrosinase presumably occurs in the trans-Golgi network as this is the first DOPA oxidase-positive compartment before the enzyme reaches the final target, the melanosome [77,78]. However, it was also reported that copper loading in the Golgi is rather inefficient [41]. There are no data concerning Trp1 and Trp2 due to the impossibility of an appropriate staining for the enzymatic activity of these proteins. In addition, the distribution of specific transport proteins and chaperones to transfer copper or zinc ions in insect cells or even mammalian fibroblasts is uncertain. Truncation of the transmembrane fragment and the C-tail in Trp1 makes the expression of the intramelanosomal domain of Trp1 possible [67], but there are many reports showing that the C-terminal tail is important for the acquisition of enzymatic activity [79]. Mutations or deletion of the entire C-terminal domain may have important consequences in the maturation process and final stability of tyrosinases and likely Trp. For instance, the C-terminal tail is needed to prevent the activation of fungal and plant tyrosinases [65,80]. The crystal structure of tyrosinase from *Aspergillus oryzae* reveals that residue F513, sited on the C-terminal end, is close to the substrate-binding site to prevent substrate access [65]. However, some cysteines sited at the C-terminal tail are also essential for Cu(II) incorporation and the tridimensional structure of that enzyme due to appropriate disulfide bridges. In that tyrosinase, copper incorporation is mediated by three Cys, a pair (C522 and C525) located on the C-terminal domain, as well as C92, which is covalently bound to H94 at the CuA site via an unusual post-translational thioether linkage. The formation of the His-Cys cross-linkage at the active site seems to be autocatalytic and concomitant with the copper incorporation [8]. Another example that illustrates the importance of the C-terminal tail in tyrosinase is the enzyme recently isolated from the marine archaeon *Candidatus Nitrosopumilus koreensis*, (*CNK*) [81]. The expression of a truncated version of this enzyme results in a form (Tyr-CNK, 1–303, fragments 304–415 removed) with lower affinity to Cu(II). The metal ions should be added to the assay buffer to maintain catalytic activity. In addition, that truncated form shows a high monophenol hydroxylase activity and is rapidly inactivated at temperatures above 35 °C, indicating that the C-terminal is important for the maintenance of the tridimensional structure and metal affinity. Maybe related to this, human tyrosinase mutations in the region close to the transmembrane fragment cause thermosensitivity [82] and results in a type of temperature-sensitive OCA [83].

C-terminal tails are surely involved in the maturation process of the tyrosinase family, as they contain differing targeting signal sequences (Figure 2) for different processing and cellular traffic [84]. There are two key signals at the C-terminal of those proteins [18,85,86]: (a) the LL dileucine motif and (b) the tyrosine-based motif, YXXB, where B can be any bulky hydrophobic residue. These two targeting signals are present in tyrosinase and Trp1, although they interact independently with adaptor proteins involved in protein sorting and final destination [37,84,85,87]. Truncated mouse tyrosinase lacking the last 27 amino acids (platinum mutation) leads to severe hypopigmentation by misrouting of the protein to the cell surface, and deletion of just the last 17 residues to eliminate the first LL motif also abolished normal transport [88].

Similarly, Tyrp1 lacking the C-terminal tail is not sorted correctly [84,89]. That tail is needed to interact with mediators of G protein signaling [90] and Rab (GTP)-binding proteins [91] during the Tyrp1 transport from the Golgi to the melanosomes [92]. In agreement with these reports, P513R is one of the mutations causing OCA3. This points out the important role of C-terminal tail of Trp1 for correct processing and folding. Truncated Trp1 species missing the C-terminal signals follow a different cellular traffic. Taking into account the different Cu(II) and Zn(II) bioavailability in cellular compartments, the metal incorporated might differ.

The number and role of Cys residues in the structure and maturation of tyrosinase family also change throughout nature. The *Streptomyces* enzymes are totally devoid of Cys, but this feature is restricted to Gram(+) bacteria [18]. Gram(−) bacteria fungal and animal tyrosinases, as well as plant catechol oxidases contain essential Cys residues. Mammalian Trps have 16–17 Cys, and 14 of them are perfectly conserved. Most of them are clustered as the N-terminal half of the protein. Twelve Cys form six disulfide bridges. The pairing positions for human Trp1 are C30–C41, C42–C65, C56–C99, C101–C110; C258–C261 and C290–C303. Crystallization of Trp1 has allowed the localization of these disulfide bridges, definitively confirming that most of the Cys form disulfide bridges as in EGF-domains for maintenance of the protein structure [66,67]. Subdomain 67–97 forms a loop located far from the active site, so that possible effects on the metal binding at the active site are unlikely, but sill important. Thus, R93C causes OCA3 due to Trp1 inactivation [93]. That Arg93 is close to four disulfide bridges in the EGF-like domain, and its replacement by Cys likely affects the disulfide pattern formation and therefore the correct folding.

The six above-mentioned disulfide bridges are conserved in the entire tyrosinase family, so that presumably they would not be related to the incorporation of different metal cofactors. However, there are at least three conserved Cys residues yet, C112, C336, C521, that have not formed disulfide bridges yet. Interestingly, human Trp2 has also two extra non-conserved Cys at positions 189 and 235. The role of those Cys residues is still unknown, but transient thiol-free-mediated bonds involved in the formation of the active site with MeA and MeB cannot be totally ruled out, similarly to the role of some Cys in *Aspergillus oryzae* tyrosinase [65].

The last important factor surely affecting the processing and acquisition of the final protein structure is the *N*-glycosylation sites and their occupancy. *N*-glycan sites are crucial for the stability, the secretory pathway and the folding of proteins. The role of *N*-glycan sites in the mammalian tyrosinase family has been extensively investigated, and this family was used as a model to study the importance of this post-translational modification [77,78,94,95,96,97,98]. Unfortunately, the conclusions are complex. On the one hand, different glycan sites on the same member of the tyrosinase family can perform distinct functions, and conserved sites on tyrosinase family paralogues can also perform different functions [97,98].

Trp1 (mouse and human) has six possible *N*-glycosylation sites (N96, 104,181, 304, 350, 385) (Figure 2). Tyrosinase has also six, but there are only three in equivalent positions. Trp1 contains five equivalent with mouse Trp2 and four with human Trp2. Mouse tyrosinase without any *N*-glycans addition fails to fold correctly [95]. On the other hand, that enzyme expressed in CHO cells is *N*-glycosylated in four of six possible sequons and correctly processed, although N111 and N161 are not occupied [95]. Treatment of mouse melanocytes with inhibitors of ER α-glucosidases prevents the association of tyrosinase with calnexin. Under these conditions, mouse tyrosinase is rapidly transported to melanosomes [96], but fails to bind copper [94]. Interestingly, native Tyrp1 is also rapidly glycosylated, as modified tyrosinase [77,78], and this might alter the acquisition of the metal cofactor.

Occupancy of the *N*-glycosylation site around the MeB [52,99] is particularly important as this is the last one at the protein sequences. The *N*-glycosylation sites are more difficult to occupy as they are closer to the C-terminal end of the protein [100]. Thus, comparison of the occupancy of this position between the entire and truncated Trp1 species might differ, linking altered length with altered *N*-glycosylation.

## 2. Final Remarks and Tentative Hypothesis

Regulation of melanogenesis in mammalian melanosome depends on three proteins, tyrosinase and two related proteins, Trp1 and Trp2. The roles of tyrosinase and Trp2 seem to be clear, according to the metal cofactor at the active site, copper and zinc, respectively. The redox properties of copper ions in tyrosinase allow for oxidizing reactions, tyrosine hydroxylase and DOPA oxidase [2]. Zn(II) has no redox properties, provided that the active site of dopachrome tautomerase has the ability to catalyze isomerization reactions, such as a keto-enol tautomerization of dopachrome to DHICA [14,49]. The metal cofactor and role of Trp1 has been uncertain for about two decades. Some discrepancies about the activity of the mouse and human enzymes have been reported [39,40], although this protein has mostly been considered as a poor tyrosinase with DHICA oxidase activity [35,36]. Due to its oxidase activity, the presence of copper at its active site has been mostly accepted. However, the recent important finding that human Trp1 expressed in insect cells has a couple of Zn(II) ions at the active site [67,68] has opened the question about the metal cofactor and function of Trp1 inside melanocytes.

Although both Trp1 and Trp2 inactivating mutations lead to moderate hypopigmentation, the phenotype of mice suffering those mutations suggests the current association of mouse Trp1 with DHICA oxidase and Trp2 with dopachrome tautomerase. Thus, Trp1 mutations lead to brown or dun pelage in the animal kingdom [101]. Human Trp1 mutations lead to OCA3 [83,93]. Concerning Trp2, *Slaty* mutation in mouse leads to reduction in the amount of eumelanin, but the content of pheomelanin is greatly increased [102,103], although the stimulation of pheomelanogenesis is not understood. Related to that, Trp2 mutations are not related to any OCA type [83]. OCA1 and OCA2 are related to tyrosinase and p-protein, respectively, and other rare OCA types from OCA4 on are related to other proteins, but not Trp2 [83]. This is in agreement with the protective role attributed to the dopachrome tautomerase activity of Trp2. Growth of *Slaty* melanocytes is severely reduced [104], due to the cytotoxic effect of decarboxylated dihydroxyindoles and subsequent indolequinones. These phenotypes suggest that Trp1 regulates the color of the obtained melanin, from black to lighter tones related to DHICA oxidase activity, but Trp2 behaves as a cytoprotective enzyme against very reactive *o*-quinone, related to the dopachrome tautomerase activity and formation of DHICA.

However, taking into account all available data discussed above, some unsolved questions arise: Does Zn-Trp1 have cellular significance in melanogenesis inside mammalian melanocytes? Does this species show dopachrome tautomerase activity? Is there an alternative Cu-Trp1 able to express DHICA oxidase activity? Are there differences between mouse and human Trp1 concerning oxidase or tautomerase activity? In summary, is Trp1 really a second oxidase, as tyrosinase, or a second tautomerase, as Trp2?

These and other questions should be answered after the interesting data reported by Lai et al. [67,68]. Cu(II) and Zn(II) are very likely incorporated into nascent proteins in different subcellular compartments and by different mechanisms due to the selectivity of chaperones and availability of both ions. Cellular toxicity of free copper ions is much higher than free zinc ions, so that intracellular availability of Zn(II) for possible binding to proteins is greater than that of copper. The tyrosinase family contains a binuclear metal binding site with the capability to load Cu(II) or Zn(II) in the three members, as was proven for tyrosinase and Trp2. The traffic and maturation of the protein through different compartments until the final destiny would determine the nature of the metal ion incorporated. The integrity of the protein should be important for appropriate traffic, *N*-glycosylation, the formation of the disulfide bridges and finally the metal acquisition at the appropriate cellular compartment. The non-melanocytic nature of the cells used for recombinant expression might also alter the maturation process in relation to melanosomes.

In addition, Zn-Trp1 expressed in insect cells can be transformed in vitro into Cu-Trp1 with residual DHICA oxidase activity [67]. Extrapolation of this pattern to melanocytes would suggest an attractive hypothesis: the capability of Trp1 accepting two different ions Cu(II) or Zn(II), according to conditions or physiological necessities. The existence of proteins susceptible to binding more than one metal ion at the active site according to the cellular conditions has not been explored so far, but it cannot be ruled out. It has been reported that alternative forms of Trp1 (gp75) can be generated during trimming reactions of the *N*-glycosylation oligosaccharide [89,97]. A speculative possibility would be the formation of two forms with different *N*-glycosylation occupancy incorporating two different metal ions, Cu(II) to acquire DHICA oxidase activity or Zn(II) to acquire tautomerase activity. In turn, the second activity would be able to act on dopachrome as a decarboxylative enzyme to yield DHI or even on a decarboxylated quinone, such as dopaminechrome, to become a second “tautomerase” activity different from Trp2. In that way, dopachrome could be transformed into DHICA or DHI depending on the action of Trp2 or Trp1, respectively. Of course, this and other possibilities require new and exciting experiments. They are probably on course as judged by the recent publications from active laboratories on this topic [9,10,67,68,70].

## Abbreviations

DHI	5,6-dihydroxyindole
DHICA	5,6-dihydroxyindole-2-carboxylic acid
EGF	Epidermal Growth Factor
IQCA	5,6-dihydroxyindole-2-carboxylic acid
MeA	Metal ion binding site A
MeB	Metal ion binding site B
OCA	Oculocutaneous albinism
Trp	Tyrosinase-related protein
